# A novel QTc–RR differential biomarker for the early assessment of autonomic dysfunction in type 2 diabetes

**DOI:** 10.3389/fendo.2026.1828837

**Published:** 2026-04-30

**Authors:** Xuwei Liao, Shanglin Yang, Hsientsai Wu, Yuyang Lin, Hongbin Zhou, Juin J. Liou

**Affiliations:** 1 School of Electrical and Information Engineering, North Minzu University, Yinchuan, Ningxia, China; 2 Microelectronics and Solid-State Electronics Device Research Center, North Minzu University, Yinchuan, Ningxia, China

**Keywords:** aging, autonomic dysfunction, entropy analysis, heart rate variability, QTc interval–RR interval differential, type 2 diabetes mellitus

## Abstract

**Purpose:**

Prolonged QT intervals are clinically relevant in patients with type 2 diabetes mellitus (T2DM). However, accurately detecting the Q and T points in electrocardiogram (ECG) signals remains challenging owing to the variability in T-wave morphology, and few engineering solutions have effectively addressed this issue.

**Methods:**

We analysed 30-min Lead II ECG recordings from 88 patients with T2DM and 93 healthy controls. A novel automated algorithm was developed to accurately detect the Q and T points, accommodating five classified T-wave morphological types. Two electrophysiologists verified the accuracy of the algorithm. Following validation, the QTc intervals derived from the algorithm were compared between the groups. Patients with T2DM exhibited significantly prolonged QTc values and a higher incidence of long QT patterns. A new parameter, RQT_diff_, the difference between the QTc and RR interval (RRI), was introduced and compared with the conventional RRI in assessing heart rate variability (HRV).

**Results:**

The proposed method accurately detected the Q and T points across diverse ECG morphologies and classified the T-wave patterns. QTc values derived from this method significantly distinguished T2DM patients from controls. The novel parameter, RQT_diff_, was strongly correlated with RRI and outperformed it in HRV analysis. Receiver operating characteristic (ROC) analysis revealed that RQT_diff_ improved the area under the curve (AUCs) for the SSR and LHR indices by 27% and 13%, respectively. Additionally, logistic regression using the Baroreflex Entropy Index demonstrated better predictive performance for RQT_diff_ over RRI, with enhanced odds ratios (Exp[B]: 0.150 vs. 0.270) and slightly improved classification accuracy (64.6% vs. 63.5%).

**Conclusion:**

This interdisciplinary study presents an engineering-based solution for robust Q- and T-point detections. The novel RQT_diff_ metric offers physiologically meaningful insights beyond the traditional RRI and enhances HRV analysis, particularly in populations with T2DM and long QT patterns.

## 1 Introduction

Heart rate variability (HRV) had long been recognized as a critical biomarker for assessing the function of the autonomic nervous system (ANS), offering valuable insights into the balance between sympathetic and parasympathetic activity (1). Through quantifying the time variations between consecutive heartbeats (RR intervals (RRIs)), HRV reflects the body’s adaptive responses to both internal and external stressors, including physiological changes, environmental stimuli, and disease processes (2). In clinical settings, HRV had been used as a valuable tool for assessing cardiovascular health, particularly in aging populations and individuals with metabolic disorders such as type 2 diabetes mellitus (T2DM) (3).

The predictive value of HRV for cardiovascular risk is well established, with lower HRV being linked to an increased risk of mortality and morbidity in various populations, including those with diabetes and the elderly (4). However, traditional linear HRV metrics based on statistical measures, such as the standard deviation of RRI or root mean square of successive differences, often fail to capture the complexity of ANS dysfunction in these populations. The challenge of early detection is compounded by the complex metabolic disturbances in aging and T2DM, which often lead to subtle autonomic dysfunctions that elude conventional RRI-based metrics (5). This limitation has prompted the development of more advanced methods for analyzing HRV, with entropy-based indices emerging as promising tools for providing a more nuanced understanding of autonomic regulation (6).

Entropy-based measures, such as multiscale entropy (MSE) and cross-entropy techniques, have gained attention due to their ability to quantify the irregularity and complexity of heart rate dynamics across multiple time scales (7, 8). Unlike traditional linear methods, which focus on short-term variability, entropy-based indices offer a comprehensive assessment of the heart’s adaptive responses through analyzing patterns over longer periods. MSE, in particular, has been demonstrated to have utility in uncovering autonomic changes that could be obscured when using conventional methods, especially in the context of chronic diseases such as T2DM (9). Furthermore, the baroreflex entropy index (BEI), a novel metric derived from the RRI, presented correlations with glycemic markers such as glycosylated hemoglobin (HbA1c) and fasting glucose, providing insights into the interplay between metabolic control and autonomic function (10).

Despite these advances, entropy-based HRV analysis using RRI still faces limitations when applied in aging and T2DM populations. These groups often exhibit overlapping symptoms of metabolic dysfunction and autonomic dysregulation, making it challenging to distinguish between healthy aging and the early stages of disease (11). As a result, researchers have explored alternative physiological parameters for entropy-based HRV analysis to improve the diagnostic sensitivity and specificity in vulnerable populations (12). Studies have evaluated amplitude variations in arterial pulse signals, surface electromyographic signals, crest time fluctuations in photoplethysmographic signals, trunk acceleration time-series, and pulse wave velocity time-series (13, 14). These approaches aim to capture a broader range of physiological dynamics, offering a more holistic view of autonomic function. Recent investigations into the QT interval—a measure of the time from the start of the Q wave to the end of the T wave in the heart’s electrical cycle—have suggested that it could offer additional insights into autonomic regulation, particularly in populations with metabolic and cardiovascular disorders. QT interval prolongation is a common feature of T2DM, which had been associated with increased cardiovascular risk and mortality in this population (15). Notably, the QT interval reflects both the depolarization and repolarization phases of the cardiac cycle, integrating information about the electrical and mechanical aspects of heart function (16). A proof-of-concept study (17) demonstrated a positive predictivity of 90% for T-waves in arrhythmic ECG signals. However, accurately detecting the Q and T points in ECG signals remains challenging owing to the variability in T-wave morphology in patients with type 2 diabetes mellitus (T2DM). Notably, distinct differences were reported in non-linear heart rate variability (HRV) measures between patients with long QT syndrome and healthy individuals, underscoring the potential value of QT interval data in complementing traditional HRV-based analysis (18). However, despite the well-documented association between T2DM and prolonged QT intervals corrected for heart rates (19, 20), no studies have explored the combined use of QTc and RRI for entropy-based HRV analysis.

Given the limitations of RRI-based entropy metrics, this study focused on the potential of the QTc interval–RR interval differential (RQT_diff_) as a novel parameter for enhancing entropy-based HRV analysis. The introduction of RQT_diff_—representing the temporal difference between the total cardiac cycle (RRI) and the corrected electrical systole (QTc). This parameter approximates the diastolic interval, a period highly sensitive to autonomic regulation that reflects the heart’s reserve for subsequent mechanical initiation, particularly in individuals with prolonged QT intervals, such as those with T2DM (21). The integration of RQT_diff_ into HRV analysis appears to be particularly relevant for aging and T2DM populations, where subtle autonomic dysfunction often precedes more overt cardiovascular complications. T2DM, for instance, has been associated with an elevated risk of both macrovascular and microvascular complications, which are often preceded by autonomic dysfunction (22, 23). Early detection of autonomic changes is crucial for preventing the progression of these complications and optimizing patient outcomes. Traditional HRV metrics, while useful, are not sufficiently sensitive to detect these early changes, particularly in populations with complex metabolic and autonomic profiles (24). Therefore, this study aimed to evaluate the utility of RQT_diff_ in refining entropy-based HRV analysis, with a specific emphasis on its application in aging and T2DM populations. Through comparing the performance of entropy metrics—such as small-scale MSE (MSE_SS_) and the BEI—calculated using both RRI and RQT_diff_, this study sought to assess the added value of incorporating QTc data into HRV analysis. The results indicate its potential to enhance the sensitivity of entropy-based HRV metrics, improving the early detection of autonomic dysfunction and informing clinical interventions for at-risk populations.

## 2 Materials and methods

### 2.1 Study population

#### 2.1.1 Inclusion and exclusion criteria

*Inclusion Criteria*: A total of 88 participants with a diagnosis of T2DM were selected based on their HbA1c levels exceeding 6.5%. These individuals were randomly recruited from the diabetic outpatient clinic at Hualien Hospital. The control group consisted of 93 healthy middle-aged to elderly individuals, recruited through a health screening program at the same hospital. Controls were screened to confirm the absence of T2DM. All participants in both groups displayed sinus rhythm on electrocardiogram (ECG).
*Exclusion Criteria*: The study excluded professional athletes. Participants with a history of cardiovascular disease—including coronary artery disease, congestive heart failure, or peripheral arterial disease—were also excluded. Furthermore, individuals with inadequate Lead II ECG measurements and those currently on medications known to influence autonomic function—such as beta blockers, calcium channel blockers, or centrally acting antihypertensive agents—were not included. In addition, detailed data regarding diabetes duration and comorbid conditions were incomplete in a portion of the cohort and therefore were not included as covariates in the present analysis.

#### 2.1.2 Grouping

The study encompassed 181 participants classified into two groups: Group 1 comprised 93 asymptomatic individuals (41 men and 52 women, aged 35 to 81 years) with hemoglobin A1c levels below 6.5%, while Group 2 included 88 individuals with T2DM, characterized by HbA1c levels greater than 6.5%.

#### 2.1.3 Ethical issues, institutional review boards, and consent form

Approval for this study was obtained from the Institutional Review Boards of Hualien Hospital (Hualien City, Taiwan) and Ningxia Chinese Medicine Research Center (Yinchuan City, Ningxia Province, China) (Approval No. 2024-214) (25). Written informed consent was acquired from all participants prior to their inclusion in the study.

### 2.2 Q and T-point detection algorithm and QTc interval calculation

#### 2.2.1 Overview

**Figure 1:**
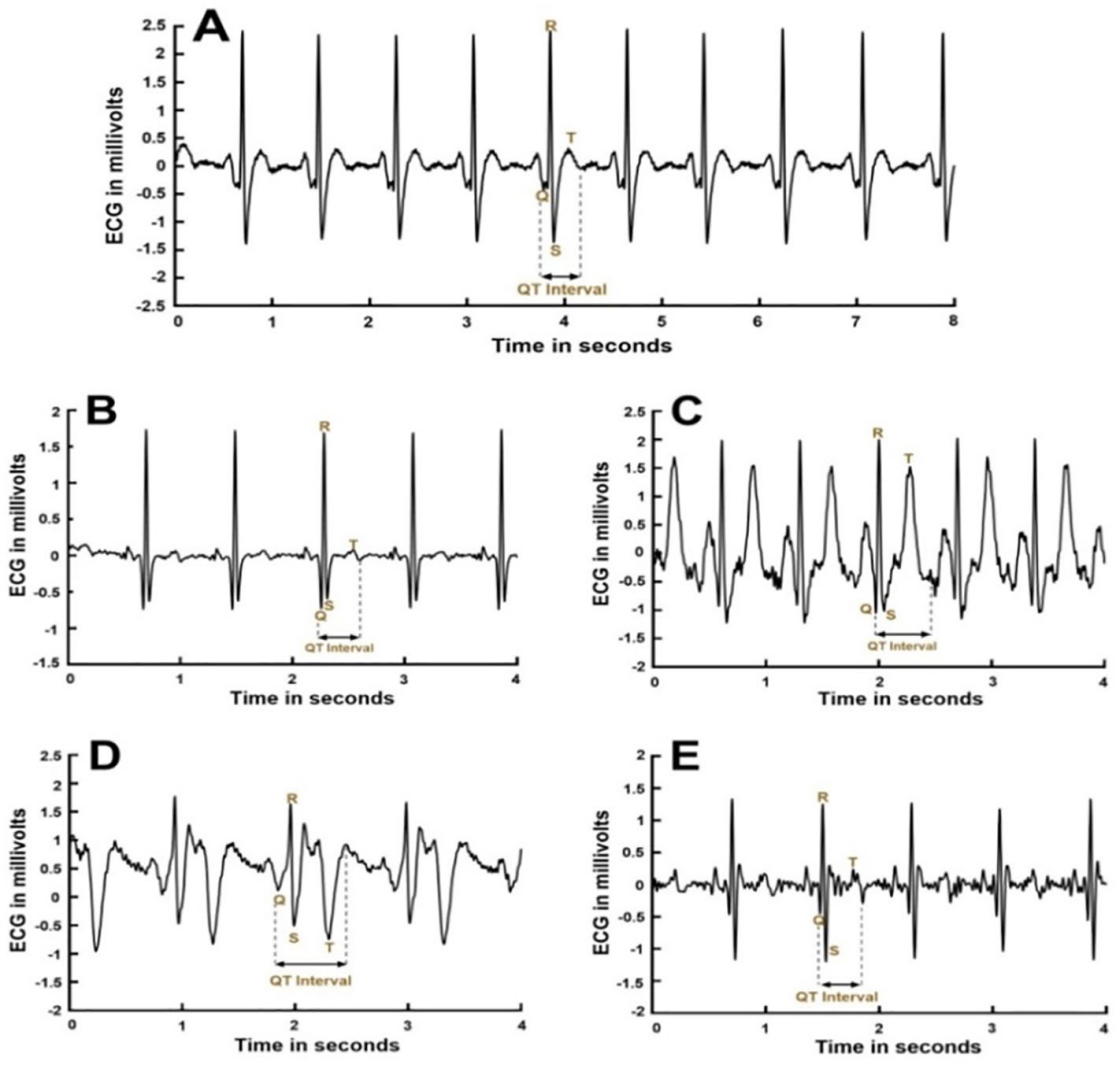
Electrocardiogram (ECG) signals and QT intervals. ECG signals from five subjects are presented: **(A)** (normal T wave), **(B)** (flattened T waves), **(C)** (peaked T waves), **(D)** (inverted T waves), and **(E)** (others). QT intervals for each subject were determined using MATLAB code based on expert-defined rules.

To accurately locate the Q and T points within the ECG signals, we developed an automated detection algorithm tailored to handle the morphological variability often observed in T2DM populations. The algorithm was designed to process 30-min Lead II ECG recordings (sampling rate: 500 Hz) and comprised the following steps:

- Preprocessing: Raw ECG signals were filtered using a bandpass filter (0.5–40 Hz) to remove the baseline drift and high-frequency noise. Subsequently, the R-peaks were detected using the enhanced Pan–Tompkins method.
- Q Point Detection: For each identified R-peak, the algorithm was searched backward within a fixed window (100–150 ms) to identify the onset of the QRS complex. The Q point was defined as the minimum deflection preceding the R-wave within this window, using a slope-based zero-crossing technique on the first derivative of the ECG signal.
- T-Point Detection: T-wave detection was more challenging owing to the morphological variability, especially in T2DM patients. The algorithm applied adaptive template matching based on local RR intervals (RRI), and dynamic thresholding. T-wave termination was defined as the point where the ECG returned to baseline or the inflection point of the descending limb, depending on the waveform morphology (**Figure 1**).
- Morphological Classification: Detected T-waves were classified into five major types—monophasic, biphasic positive-negative, biphasic negative-positive, notched, and flat—based on amplitude, polarity, and derivative features. This classification helped to adapt the detection parameters in a morphology-specific manner (17).
- Verification and Correction: All detected Q and T points were reviewed by two experienced and board-certified clinical electrophysiologists who were blinded to the clinical group assignments. Manual corrections were made in cases of algorithmic misidentification, and all manually corrected ECG annotations were independently affirmed by two experienced electrophysiologists again. Any discrepancies between the two reviewers were resolved through consensus discussion to ensure annotation consistency and reduce potential observer bias, and the corrected dataset was used for further HRV and QTc–RR differential analyses.

To determine the QT interval from Lead II ECG recordings after Q/T finding, the following steps were taken:

1. Identification of the QT Interval: the QT interval was defined as the time from the onset of the QRS complex to the end of the T wave, representing ventricular depolarization and repolarization, respectively.
2. Measurement of the QT Interval: The duration of the QT interval was measured from the beginning of the QRS complex to the end of the T wave using calipers or digital tools. Care was taken to include the Q wave, when present, ensuring accurate measurements from the onset of the QRS complex.
3. QTc Calculation: The QTc using the Bazett formula: QTc = QT interval/√RRI (in seconds), where the RRI represents the time between consecutive R waves. Other formulas, such as those of Fridericia and Hodges, were used when appropriate for specific circumstances.
4. Interpretation of QTc: The QTc was compared with normal reference ranges, adjusted for the patient’s age, sex, and relevant clinical factors. Prolongation of the QTc is associated with increased risks of arrhythmias, including torsades de pointes.

Accurate QT interval measurement is critical, requiring expertise in ECG interpretation. Errors in measurement can have serious clinical implications; therefore, the evaluation was conducted by qualified professionals, such as cardiologists or experienced technicians, following established guidelines (26).

**Figure 2:**
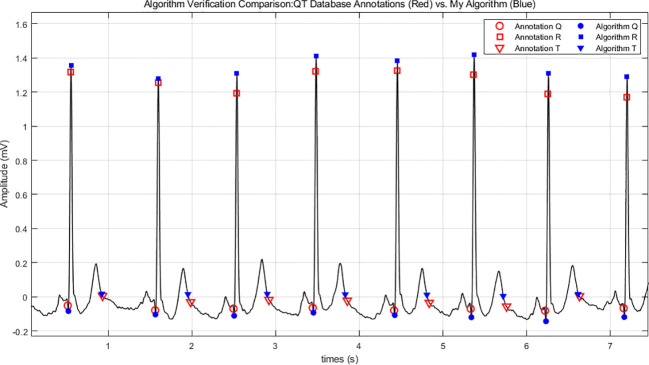
Comparison of fiducial point detection between expert annotations and the custom algorithm. A representative ECG segment from the PhysioNet QT Database. Red markers represent expert annotations (Q_on_, R_peak_, T_off_), and blue markers represent detected points by our custom algorithm. The close spatial convergence demonstrates high delineation accuracy.

To ensure the technical validity of the extracted features, the automated algorithm was rigorously validated against the authoritative PhysioNet QT Database (QTDB) benchmark. The validation was performed on high-quality cardiac cycles (defined as having a T-wave amplitude >15% of the R-peak). For each cycle, the detected points (Q_on_, R_peak_, T_off_) were compared with expert annotations to calculate interval errors. Representative delineation results are illustrated in **Figure 2**.

#### 2.2.2 Novel ECG parameters for HRV

The RRI represents the interval between successive R waves and reflects the overall duration of the cardiac cycle. In the present study, we introduce the QTc–RR interval differential (RQT_diff_) as a derived ECG-based parameter defined as the difference between the RR interval (RRI) and the heart rate–corrected QT interval (QTc) as Equations 1–3, where QTc is calculated using Bazett’s formula.

Conceptually, RQT_diff_ combines information from two established ECG measurements: RRI, which reflects heart rate dynamics, and QTc, which represents ventricular depolarization and repolarization duration adjusted for heart rate. As such, RQT_diff_ can be interpreted as a composite temporal index reflecting the relationship between cardiac cycle length and ventricular electrical activity.

**Figure 3:**
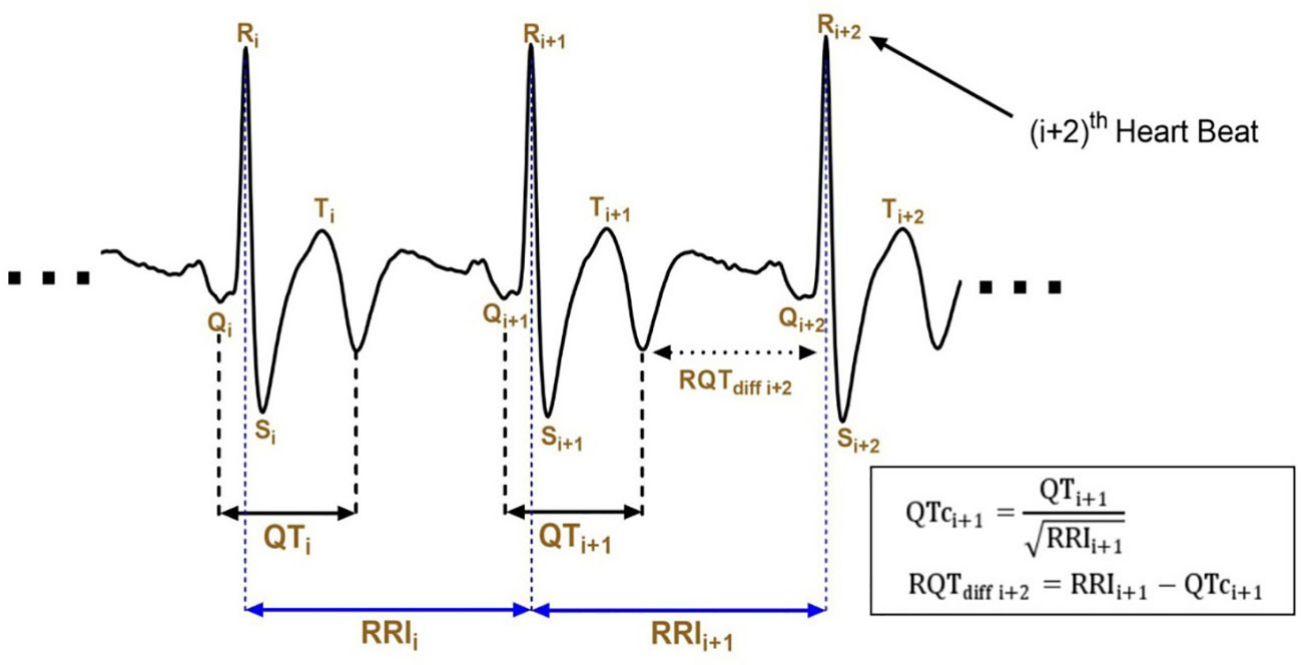
QTc interval–RR interval differential (RQT_diff_) parameter calculation. ECG signals were captured at a sampling rate of 500 Hz for 2000 heartbeats. To illustrate the RQT_diff_ parameter, only three consecutive R waves (R_i_, R_i+1_, R_i+2_) and two RR intervals (RRI_i_, RRI_i+1_) are depicted. The QT interval (QT_i+1_) is determined as shown in **Figure 3**, between R waves R_i+1_ and R_i+2_. The corrected QT interval (QTc_i+1_) is calculated using Bazett’s formula: QTc_i+1_ = QT_i+1_/√RRI_i+1_. The RQT_diff_ parameter (RQT_diff i+2_) is the difference between the RR interval (RRI_i+1_) and the corrected QT interval (QTc_i+1_): RQT_diff i+2_ = RRI_i+1_ − QTc_i+1_.

From a signal analysis perspective (**Figure 3**), RQT_diff_ corresponds to the remaining portion of the cardiac cycle after accounting for ventricular depolarization–repolarization duration. Rather than representing a distinct physiological compartment or conduction process, it serves as a derived feature that integrates multiple aspects of cardiac timing.

The rationale for introducing RQT_diff_ in this study is to explore whether combining RRI and QTc into a single parameter can enhance the sensitivity of HRV analysis, particularly in populations with subtle autonomic dysfunction such as aging individuals and patients with type 2 diabetes mellitus. Therefore, RQT_diff_ is used as a complementary index alongside conventional RRI-based HRV metrics in subsequent entropy and statistical analyses.

(1) $$\left\{{\text{RQT}}_{\text{diff}}\right\}\equiv \left\{\text{QTc}-\text{RR differrence}\right\};\text{ QTc is QT interval corrected for heart rate}.$$

(2) ={RRI−QTc},

(3) ={RRI}−{QT}/RRI, where RRI's unit is seconds.

#### 2.2.3 Q and T point detection algorithm

To enable accurate extraction of QT intervals from Lead II ECG signals, we developed a custom signal processing algorithm for automatic detection of Q and T points. The method begins with preprocessing, where the raw ECG signal is filtered using a bandpass filter (0.5–40 Hz) to remove baseline wander and high-frequency noise. R peaks are then detected using a modified Pan–Tompkins algorithm, providing temporal anchors for subsequent Q and T point localization.

For Q point detection, the algorithm defines a fixed window (e.g., 150 ms) prior to each R peak and identifies the local minimum within this window, aided by zero-crossings in the first derivative to improve accuracy. T point detection is more complex due to morphological variability in T-wave patterns, particularly in diabetic populations. A dynamic window (typically 200–500 ms following the R peak) is used to search for the T wave. The T wave peak is first identified, and the T offset is then determined using a combination of methods: the threshold-based return-to-baseline approach, the tangent method on the maximum downslope, and the second derivative inflection point method.

To accommodate varying T-wave morphologies, detected waveforms are automatically classified into five categories—monophasic, biphasic (positive-negative or negative-positive), notched, and flat—based on amplitude, polarity, and slope features. Detection parameters are adjusted accordingly to improve robustness. All Q and T annotations were independently reviewed and validated by two clinical electrophysiologists. These fiducial points were then used to compute QT intervals and correct them for heart rate (QTc), forming the basis for the QTc–RR interval differential (RQTdiff) used in subsequent HRV and statistical analyses. The detailed step-by-step pseudocode for the Q and T-point detection and morphology classification, along with the complete MATLAB scripts, are provided in the **Supplementary Material**.

### 2.3 HRV indices: RRI vs. RQT_diff_

#### 2.3.1 Low-to-high frequency power ratio and sympathetic stress ratio

##### 2.3.1.1 LHR: a linear HRV index

The LHR is a commonly used linear HRV index that reflects the balance between sympathetic and parasympathetic nervous system activities. It is defined as the ratio of low-frequency power (LFP; 0.04–0.15 Hz) to high-frequency power (HFP; 0.15–0.40 Hz) and is calculated as LHR = LFP/HFP. A higher LHR value indicates a predominance of sympathetic over parasympathetic modulation, a condition often linked to an elevated cardiovascular risk (27).

##### 2.3.1.2 Poincaré plot index (SSR): a hybrid HRV index

The SSR, derived from the Poincaré plot, is considered a hybrid HRV index that bridges linear and non-linear analyses. It is computed as the ratio of SD1 to SD2, where SD1 reflects short-term (instantaneous) RRI variability and SD2 represents long-term variability. These values correspond to the standard deviations of points perpendicular and parallel to the line of identity on the Poincaré plot, respectively. SSR is calculated as SSR = SD1/SD2, providing a concise measure of the autonomic balance between fast and slow heart rate fluctuations (28).

#### 2.3.2 MSE_SS_

MSE is a widely utilized method in HRV analysis that extends traditional entropy measures through assessing the complexity of physiological time-series across multiple time scales. The process begins with coarse graining, where the original time-series is systematically divided into shorter segments representing different scales. At each scale, a new time-series is generated through averaging consecutive data points. Entropy is then calculated for each of these coarse-grained time-series, providing insight into the system’s complexity not only at short but also at longer temporal scales. This method has been shown to be valuable for detecting subtle changes in autonomic function, particularly in aging or diabetes populations, where cardiovascular control mechanisms may be compromised (7, 8). MSE analysis in cardiovascular studies investigates physiological complexity through the following steps: (1) coarse graining, which captures system dynamics across multiple scales, and (2) entropy calculation for both the original signal and the coarsened series, allowing for assessment of irregularity at each scale. Age and disease states are known to decrease physiological complexity, resulting in lower MSE values. The MSESS provides important insights into the improvement in ANS function (12).

#### 2.3.3 BEI

The BEI offers another innovative approach for the assessment of autonomic function, particularly in populations with T2DM. This index, derived from Lead II ECG, quantifies the complexity of baroreflex activity, which regulates blood pressure via autonomic feedback mechanisms. The BEI is calculated by evaluating the entropy of heart rate fluctuations in relation to baroreflex sensitivity, providing insight into the integrity of autonomic control. Recent research has demonstrated its clinical relevance, particularly in T2DM populations, where autonomic dysfunction is prevalent. For instance, the study of Yang et al. (10) established the efficacy of BEI in differentiating between patients with T2DM and healthy controls, highlighting its potential as a valuable tool for the early detection of autonomic impairments.

### 2.4 Statistical analyses

T wave classifications in Lead II ECG for each subject were determined based on the exact QT intervals calculated using MATLAB, following expert-defined rules. A graphical representation was created to illustrate the heart rate variations, QTc, and RQTdiff across 1000 beats, highlighting the effects of aging and T2DM. The correlation coefficients between these parameters showed minimal variation due to aging and T2DM. The comparison of HRV performance between the BEI and MSESS is presented in **Table 1**, with the results expressed as the mean ± standard deviation (SD). For MSESS and BEI, data are also presented as mean ± standard error of the mean, calculated as SD/√N. Statistical analyses were conducted using the SPSS software (version 14.0, IBM Corp., Chicago, IL, USA) and MATLAB (R2020b, MathWorks Inc., Natick, MA, USA) Ternary diagrams were constructed to represent the relationships between HbA1c, fasting plasma glucose (FPG), and entropy-based indices, along with RQTdiff. Additionally, receiver operating characteristic (ROC) curve analysis was conducted to evaluate the efficacy of ECG parameters and entropy-based indices in distinguishing healthy individuals from those with T2DM or age-related autonomic dysfunction.

**Table 1:** Anthropometric, biochemical, RQT_diff_, and entropy parameters of the study groups (Group 1: healthy middle-aged participants; Group 2: patients with type 2 diabetes mellitus).

Parameter	Group 1 Group 2		*p-*value
Number (male/female) Age (years)	93 (41/52) 50.56 ± 12.35	88 (36/52) 62.77 ± 10.30	N/A *p* < 0.001^*^
WC (cm)	84.04 ± 11.64	93.32 ± 11.60	*p* < 0.001^*^
BMI (kg/m^2^)	24.59 ± 3.64	27.24 ± 5.24	*p* < 0.001^*^
SBP (mmHg)	118.53 ± 13.79	127.16 ± 15.94	*p* < 0.001^*^
DBP (mmHg)	75.53 ± 9.83	77.58 ± 10.51	*p* = 0.176
HR (beats/min)	68.62 ± 10.05	71.33 ± 9.83	*p* = 0.069
HDL (mg/dL)	47.63 ± 17.31	41.91 ± 9.47	*p* = 0.007^*^
LDL (mg/dL)	112.20 ± 34.20	119.74 ± 37.46	*p* = 0.189
Cholesterol (mg/dL)	175.49 ± 43.47	190.73 ± 46.27	*p* = 0.024^*^
Triglyceride (mg/dL)	101.41 ± 53.13	141.61 ± 59.76	*p* < 0.001^*^
HbA1c (%)	5.90 ± 0.50	7.86 ± 1.56	*p* < 0.001^*^
FPG (mg/dL)	99.97 ± 18.68	161.75 ± 45.74	*p* < 0.001^*^
RRI (ms)	894.51 ± 142.20	856.75 ± 116.56	*p* = 0.053
RQT_diff_ (ms)	422.71 ± 177.56	349.88 ± 143.81	*p* = 0.003^*^
QTc (ms)	471.79 ± 68.58	506.87 ± 73.87	*p* < 0.001^*^
MSE_SS_ (RRI)	1.87 ± 0.33	1.74 ± 0.38	*p* = 0.015^*^
LHR (RRI)	1.38 ± 0.25	1.31 ± 0.29	*p* = 0.872
SSR (RRI)	0.70 ± 0.14	0.70 ± 0.17	*p* = 0.994
BEI (RRI)	1.52 ± 0.50	1.24 ± 0.50	*p* < 0.001^*^
MSE_SS_ (RQT_diff_)	2.00 ± 0.25	1.90 ± 0.33	*p* = 0.024^*^
LHR (RQT_diff_)	1.17 ± 0.23	1.04 ± 0.27	*p* < 0.001^*^
SSR (RQT_diff_)	0.79 ± 0.11	0.84 ± 0.12	*p* = 0.002^*^
BEI (RQT_diff_)	1.45 ± 0.40	1.26 ± 0.30	*p* < 0.001^*^

The total number of test subjects was 181. Group 1: middle-aged asymptomatic individuals; Group 2: middle-aged patients with T2DM. Abbreviations: WC, waist circumference; BMI, body mass index; SBP, systolic blood pressure; DBP, diastolic blood pressure; PP, pulse pressure; HR, heart rate; HDL, high-density lipoprotein; HbA1c, glycosylated hemoglobin; FPG, fasting plasma glucose; MSE_SS_ and MSE_LS_, small-scale and large-scale multiscale entropy index; BEI, baroreflex entropy index; QTc, corrected QT interval (calculated as QT/√RRI); RQT_diff_, RRI minus QTc. Group comparisons were conducted using an unpaired t-test. Non-normally distributed parameters, including WC, HDL, HbA1c, and RRI, were analyzed with non-parametric tests (Mann–Whitney U-test and Wilcoxon signed-rank test). all results are consistently presented as mean ± standard deviation (SD). The p-values* indicate statistical differences in group means.

## 3 Result

### 3.1 T-wave classification in lead II ECG

In this study, we employed refined Lead II ECGs (10) to obtain 30-minute recordings, which were then analyzed to calculate both small and large time-scale MSE indices and the BEI as measures of HRV. These indices were used to assess autonomic function in healthy middle-aged adults and patients with T2DM, in order to examine their clinical associations. The recorded ECG signals, displaying various T wave morphologies (28), are shown in **Figure 1**. The QT interval—a key marker of ventricular depolarization and repolarization—was determined for each participant using the MATLAB software based on expert-defined protocols. To account for HRV, the QT interval was corrected to QTc using the Bazett formula (QTc = QT interval/√RRI, in seconds), with the RRI measured between consecutive R waves (**Figure 1**).

Before data analysis, all test subjects were categorized into five distinct T wave types based on their Lead II ECG signals. The QT intervals were determined using MATLAB code based on expert-defined rules, as depicted in **Figure 1**. In Group 1, 70 out of 93 subjects (75.3%) displayed a normal T wave (Type A in **Table 2**), while, in Group 2, 59 out of 88 subjects (67.0%) exhibited a normal T wave. In contrast, abnormal T waves (Types B, C, D, and E in **Table 2**) were observed in 23 subjects (24.7%) in Group 1 and 29 subjects (33.0%) in Group 2.

**Table 2:** Distribution of normal and abnormal T waves in ECG for subjects in groups 1 and 2.

ECG T wave types	Group 1	Group 2
A	70	59
B	8	9
C	2	5
D	10	12
E	3	3
Total	93	88

Type A, subjects with normal T waves; Type B, subjects with flattened T waves; Type C, subjects with peaked T waves; Type D, subjects with inverted T waves; and Type E, subjects with other abnormal T waves. Clinically, Type A represents a normal ECG T wave, while Types B, C, D, and E indicate various abnormal ECG T waveforms. Group 1 consists of middle-aged asymptomatic individuals, while Group 2 comprises middle-aged patients with type 2 diabetes mellitus (T2DM). In Group 1, 70 of the 93 subjects (75.3%) had a normal T wave, whereas, in Group 2, 59 of the 88 subjects (67.0%) exhibited a normal T wave.

### 3.2 Algorithm validation results

The large-scale benchmark validation across high-fidelity cycles in the QTDB demonstrates high precision for both RRI and QTI (**Table 3**). The algorithm achieved a Positive Predictive Value (PPV) of 95.80% and a Median Error of 0.01 ms (SD: 6.64 ms) for RRI, perfectly aligning with the expert gold standard for baseline heart rate detection.

**Table 3:** Performance metrics of the automated detection algorithmagainst the PhysioNet QT database benchmark.

	PPV	Median	Mean	SD
RRI	95.80%	0.01 ms	0.05 ms	6.64 ms
QTI	84.65%	-24.00 ms	-19.77 ms	27.42 ms

Metrics are reported for R-R Interval (RRI) and Q-T Interval (QTI) based on high-quality cardiac cycles (T-wave amplitude >15% of R-peak). PPV, Positive Predictive Value; SD, Standard Deviation.

For the critical Q-T Interval (QTI), the algorithm exhibited minimal bias with a Median Error of -24.00 ms and a remarkably low Standard Deviation of 27.42 ms. This level of precision is well within acceptable clinical limits for automated delineation and strongly validates the integrity of the temporal anchors used to construct the subsequent entropy-based parameters.

### 3.3 QTc and RQT_diff_ for test subjects

**Figure 4:**
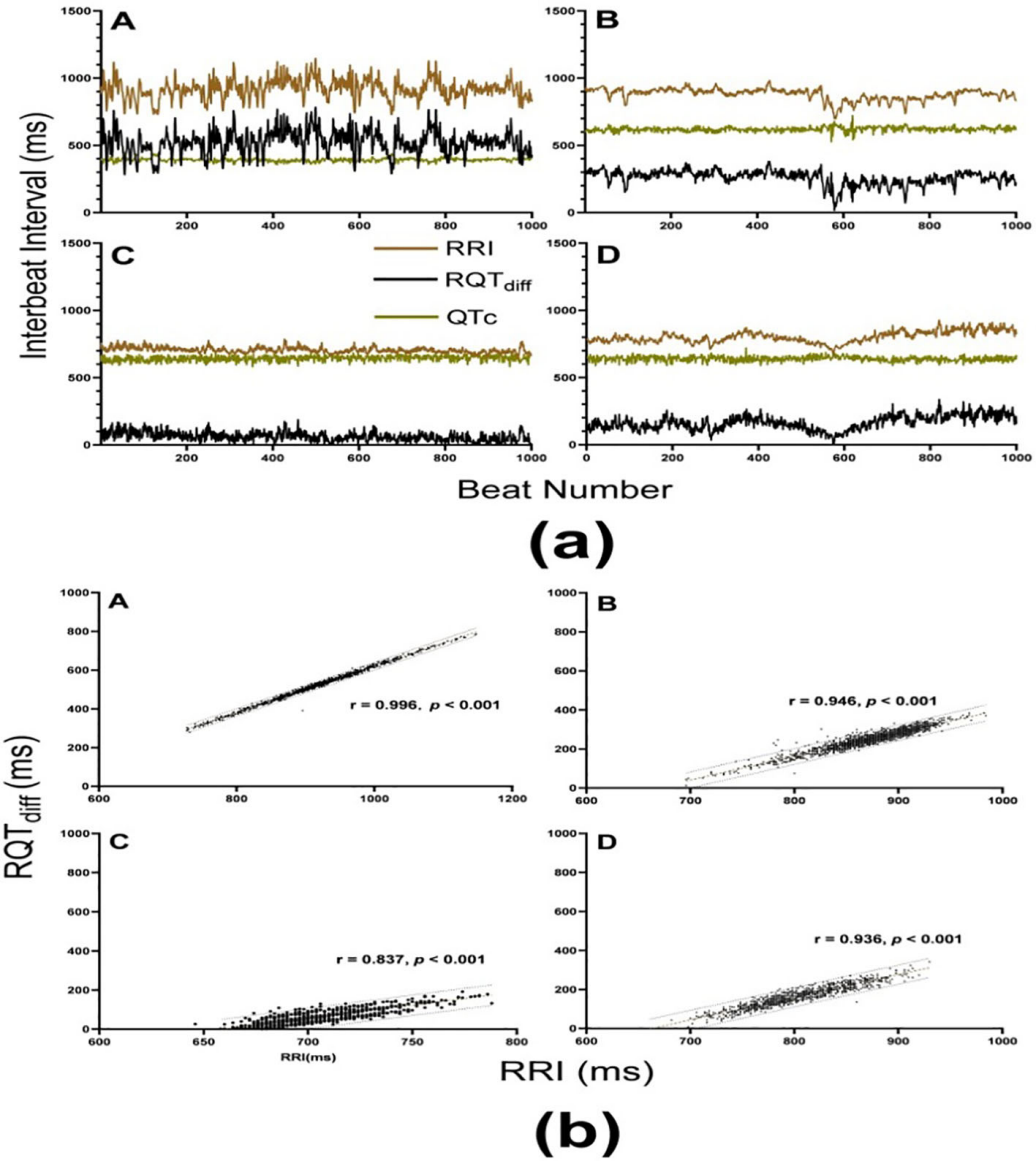
**(a)** Heart rate variations, corrected QT interval (QTc), and RQT_diff_ over 1000 beats for four subjects: A–D Subjects A and B, both healthy (subject A: male, age 22, BMI: 22.4, HbA1c: 5.5; subject B: female, age 58, BMI: 20.3, HbA1c: 6.2), show moderate fluctuations in heart rate during the recorded period. In contrast, subjects C and D, both with T2DM (subject C: male, age 28, BMI: 41.2, HbA1c: 8.3; subject D: female, age 56, BMI: 28.3, HbA1c: 8.5), exhibit distinct heart rate profiles. Subject C demonstrates a marked reduction in dynamic variability, whereas subject D presents a significantly elevated HRV. Despite these variations, all subjects display consistent patterns in their RRI and RQT_diff_, with RRI consistently greater than RQT_diff_. QTc values for all four subjects remain within a narrow range. **(b)** A strong positive correlation (all *p* < 0.001) was observed between RRI and RQT_diff_ for all four subjects shown in **(a)**. The healthy young subject A exhibited the highest positive correlation, whereas the two diabetic subjects C, D showed reduced correlation coefficients, along with a narrower range of variation in both RRI and RQT_diff_ (with lower RQT_diff_ than subjects A, B). The association between RRI and RQT_diff_ was tested using the Spearman correlation test.

Subjects A and B, both categorized as healthy individuals, served as a control group. Subject A is a 22-year-old male with a BMI of 22.4 and an HbA1c level of 5.5, indicating normal glucose metabolism. Subject B, a 58-year-old female, has a BMI of 20.3 and an HbA1c level of 6.2, which is still within the non-diabetic range. In contrast, subjects C and D had both been diagnosed with T2DM. Subject C is a 28-year-old male with a significantly higher BMI of 41.2 and an HbA1c level of 8.3, indicative of poor glycemic control. Subject D, a 56-year-old female, also has diabetes, with a BMI of 28.3 and an HbA1c level of 8.5. As shown in **Figure 4A**, all four subjects exhibited similar trends in their RRI and RQT_diff_ values, with the RRI consistently surpassing the RQT_diff_ measurements. Interestingly, the QTc for all subjects remained tightly clustered within a narrow range, suggesting a relatively stable cardiac repolarization process despite differences in metabolic and health status. The correlation analysis presented in **Figure 4B** reveals a strong positive correlation between RRI and RQT_diff_ for all four subjects, with statistical significance (*p* < 0.001) across the board. Among these, subject A—the younger and healthier male—demonstrated the strongest correlation between RRI and RQT_diff_. Conversely, subjects C and D—both of whom have diabetes—displayed a decline in the strength of this correlation, coupled with a narrower range of variation in both their RRI and RQT_diff_ measures.

### 3.4 HRV performance comparison between BEI and MSE_SS_ with RRI and RQT_diff_

The results indicated no statistically significant differences between the participants with diabetes (Group 2) and those without diabetes (Group 1), in terms of diastolic blood pressure (*p* = 0.176) and low-density lipoprotein (*p* = 0.189); see **Table 1**. Among the ECG parameters—RRI, RQT_diff_, and QTc—and their respective entropy indices, only RRI, LHR(RRI) and SSR(RRI)demonstrated non-significant differences, with *p*-values of 0.053,0.872 and 0.994, respectively.

Only three parameters from the data analysis—BEI (RRI), BEI (RQT_diff_), and QTc—showed statistically significant differences in group means based on ANCOVA (*p* < 0.05), after adjusting for covariates, including age, waist circumference (WC), body mass index (BMI), systolic blood pressure (SBP), HDL cholesterol, total cholesterol, triglycerides, HbA,1c, and fasting plasma glucose (FPG).

### 3.5 Ternary diagrams for HbA1c, FPG, and entropy-based indices with RQT_diff_ parameter

**Figure 5:**
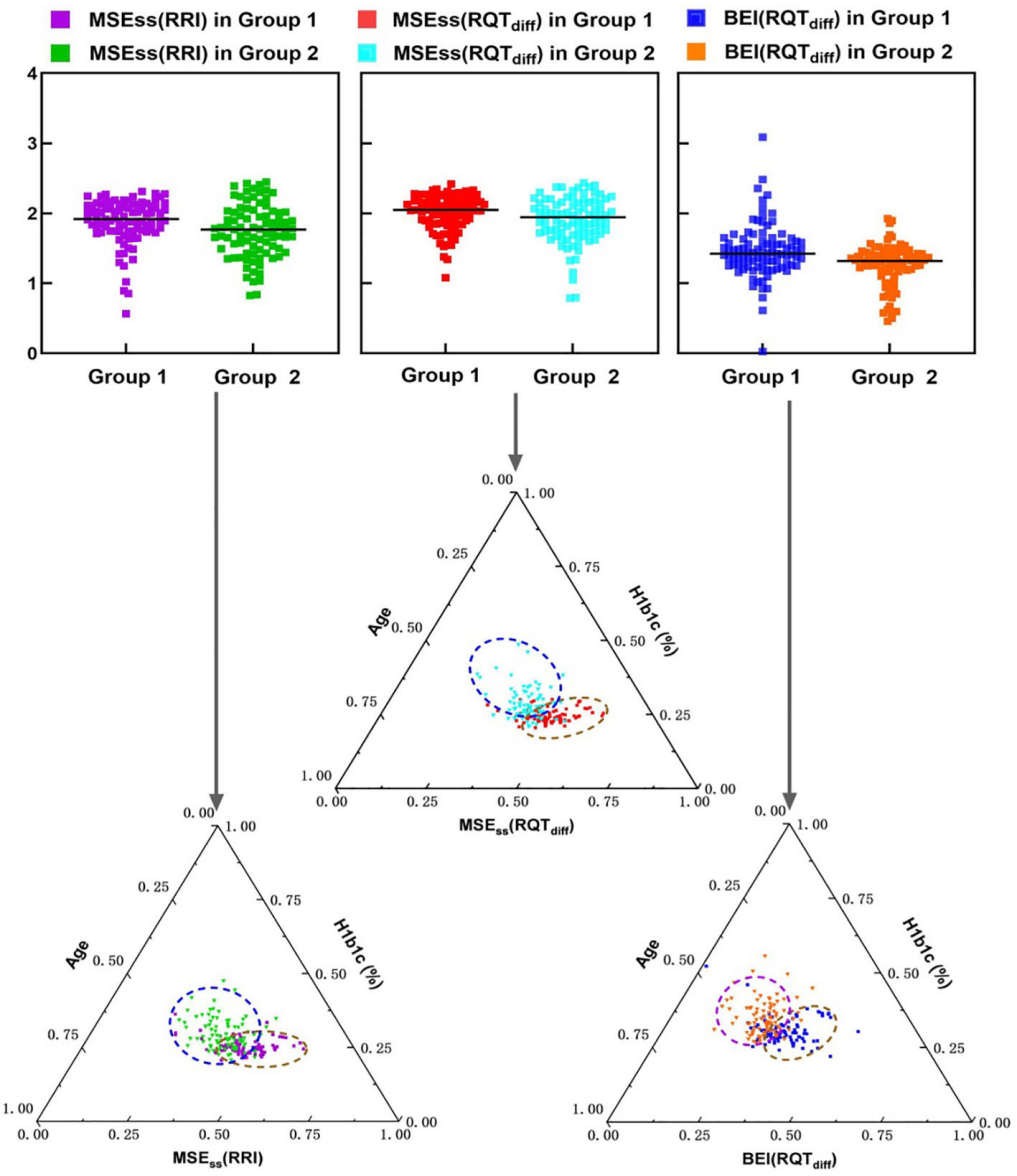
Ternary diagrams illustrate the separation ability of entropy-based indices—namely, MSE_SS_(RRI), MSE_SS_(RQT_diff_), and BEI(RQT_diff_)—between the two groups, highlighting the impact of combined glycosylated hemoglobin (HbA1c) and age. For MSE_SS_, RQT_diff_ demonstrated superior performance compared to RRI.

This study identified statistically significant differences in HbA1c, age, and the entropy-based indices MSE_SS_(RRI), MSE_SS_(RQT_diff_), and BEI(RQT_diff_) between the two groups, namely, healthy middle-aged subjects and patients with T2DM (see Section 3.1). Ternary diagrams (**Figure 5**) were used to illustrate the effectiveness of these indices in distinguishing between the groups based on the combined effects of HbA1c and age. Among the indices, BEI(RQT_diff_) and MSE_SS_(RQT_diff_) provided superior visual discrimination of group differences, when compared to MSE_SS_(RRI).

### 3.6 ROC curve analysis for HRV indices with RRI vs. RQT_diff_

**Figure 6:**
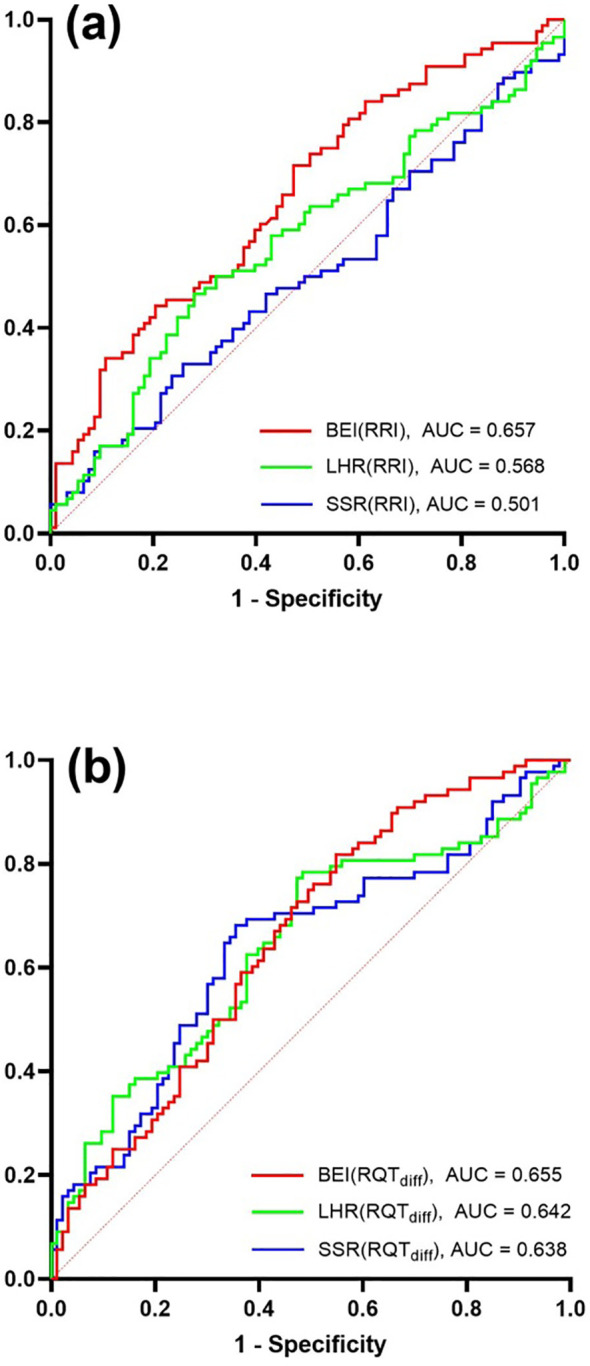
Receiver operating characteristic (ROC) curve analysis is performed to determine the optimal cutoffs for the following HRV indices—BEI, LHR, and SSR—with **(a)** RRI parameter, and **(b)** RQT_diff_ parameter for predicting T2DM. ROC analysis reveals that RQT_diff_ improved the AUCs for the SSR and LHR indices by 27% and 13%, respectively.

Receiver operating characteristic (ROC) curve analysis was conducted to evaluate the performance of the HRV indices—BEI, LHR, and SSR—in predicting T2DM using both the conventional RRI and proposed RQT_diff_ parameter. As shown in **Figure 6**, the RQT_diff_-based analysis showed higher AUC values compared with the RRI-based analysis, suggesting a potential improvement in classification performance. Specifically, the area under the curve (AUC) for SSR increased by 27% (from 0.501 to 0.638) and for LHR by 13% (from 0.568 to 0.642) compared with the RRI-based analysis (**Figure 6**). These findings demonstrate that RQT_diff_ offers enhanced discriminatory power over RRI in entropy- and frequency-domain HRV assessments for identifying T2DM risk. However, these findings should be interpreted as indicative rather than definitive due to the moderate AUC values.

### 3.7 Logistic regression analysis using HRV indices: RRI vs. RQT_diff_

To assess the predictive value of HRV indices for identifying T2DM, binary logistic regression analysis was performed using both conventional RRI-based and QTc–RR differential (RQT_diff_)-based parameters. **Table 4** summarizes the model coefficients, significance levels, odds ratios (Exp(B)), confidence intervals, model fit (Hosmer–Lemeshow test), and overall classification accuracies. The BEI showed a stronger association with T2DM when computed from RQT_diff_ rather than the RRI. Specifically, the RQT_diff_-based BEI model yielded an odds ratio of 0.150 (95% CI: 0.051–0.440, *p* = 0.001) and improved the overall classification accuracy to 64.6%, compared with 63.5% using the RRI-based model (Exp(B): 0.270, *p* < 0.001).

**Table 4:** Logistic regression analysis of type 2 diabetes with HRV indices with RRI vs. RQT_diff._.

Parameter	B	Sig.	Exp(B)	95% CI for Exp(B)	*p* value of Hosmer- Lemeshow test	Overall percentage
BEI (RRI)	-1.308	< 0.001	0.270	0.131 - 0.599	0.359	63.5%
BMI	0.158	< 0.001	1.171	1.077 - 1.274		
Constant	-2.335	0.042	0.016	N/A		
MSE_SS_(RRI)	-1.280	0.006	0.278	0.112 - 0.690	0.966	62.4%
BMI	0.152	< 0.001	1.164	1.077 - 1.259		
Constant	-1.675	0.184	0.187	N/A		
LHR (RRI)	-0.959	0.102	0.383	0.043 - 0.413	0.089	62.4%
BMI	0.141	< 0.001	1.152	1.051 - 1.124		
Constant	-2.414	0.064	0.089	N/A		
SSR (RRI)	-0.341	0.738	0.711	0.096 - 5.262	0.149	58.6%
BMI	0.145	< 0.001	1.156	1.069 - 1.250		
Constant	-3.576	0.003	0.028	N/A		
BEI (RQT_diff_)	-1.899	0.001	0.150	0.051 - 0.440	0.441	64.6%
BMI	0.155	< 0.001	1.168	1.075 - 1.270		
Constant	-1.483	0.237	0.227	N/A		
MSE_SS_(RQT_diff_)	-1.517	0.008	0.219	0.072 - 0.672	0.426	66.3%
BMI	0.157	< 0.001	1.169	1.080 - 1.266		
Constant	-1.154	0.416	0.315	N/A		
LHR (RQT_diff_)	-2.104	0.001	0.122	0.035– 0.431	0.400	65.2%
BMI	0.147	< 0.001	1.158	1.071 - 1.252		
Constant	-1.529	0.210	0.217	N/A		
SSR (RQT_diff_)	4.117	0.003	61.357	4.036 – 932.740	0.084	64.1%
BMI	0.141	< 0.001	1.152	1.066 - 1.245		
Constant	-7.079	< 0.001	0.001	N/A		

B—Coefficient; Sig.—*p*-value< 0.05 was considered statistically significant for the test parameter; OR, odds ratio, Exp(B); CI, confidence interval; WC, waist circumference; RRI R-R interval; RQT_diff_, the novel ECG parameter for HRV in Eq.(3); LHR, the low-to-high frequency power ratio (27); SSR, Poincaré plot index with mean lag 1 to 10 (28); BEI, baroreflex entropy index (10); The fitted logistic regression models were all with the same results using different types in SPSS. All models were adjusted for BMI as a covariate. Confidence intervals (95% CI) and goodness-of-fit statistics (Hosmer–Lemeshow test) are reported to ensure model robustness. The odds ratio for SSR (RQTdiff) is accompanied by a wide confidence interval, indicating variability and potential instability of the estimate.

Similarly, the multiscale entropy steady-state (MSESS) showed improved predictive power when derived from RQT_diff_. The RQT_diff_-based model achieved an Exp(B) of 0.219 (*p* = 0.008) with a classification accuracy of 66.3%, outperforming the RRI-based model (Exp(B): 0.278, *p* = 0.006; accuracy: 62.4%). For the LHR index, RQT_diff_ demonstrated superiority with a significant odds ratio of 0.122 (95% CI: 0.035–0.431, *p* = 0.001) and improved accuracy (65.2%), whereas the RRI-based model was not statistically significant (*p* = 0.102; accuracy: 62.4%).

Notably, SSR based on RQT_diff_ revealed a dramatic increase in odds ratio (Exp(B): 61.357, *p* = 0.003) and achieved an overall classification accuracy of 64.1%, However, the corresponding odds ratio was large (OR = 61.357) and accompanied by a wide 95% confidence interval (4.036–932.740), indicating substantial variability in the effect estimate. This may be due to scaling effects and the distributional characteristics of the SSR variable. Therefore, this finding should be interpreted as exploratory rather than as a precise quantitative risk estimate. The RRI-based SSR model was not significant (*p* = 0.738; accuracy: 58.6%) (**Table 4**).

**Figure 7:**
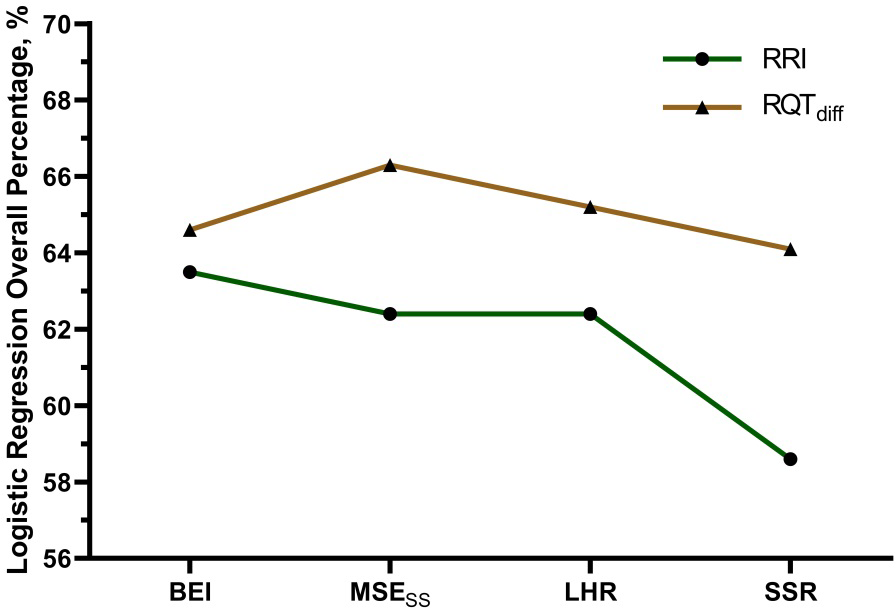
Comparison of overall classification accuracy in logistic regression analysis for predicting type 2 diabetes mellitus using four HRV indices—Baroreflex Entropy Index (BEI), small-scale Multiscale Entropy (MSE_ss_), Low-to-High Frequency Ratio (LHR), and Poincaré plot index (SSR)—with two different input parameters: R–R interval (RRI) and QTc–RR differential (RQT_diff_). Across all indices, the models incorporating RQT_diff_ consistently achieved higher classification performance than those using the RRI.

**Figure 7** presents a comparison of the overall classification accuracy in the logistic regression analysis of T2DM using four HRV indices—BEI, MSEss, LHR, and SSR—with two different input parameters: RRI and RQT_diff_. The results demonstrated that replacing the RRI with the RQT_diff_ consistently improved the predictive performance across all HRV indices, highlighting the added value of the RQT_diff_ parameter in diabetes risk classification.

## 4 Discussion

This study proposes the RQT_diff_, as depicted in **Figure 3**, as a promising novel parameter for enhancing entropy analysis in HRV assessments, particularly within aging and T2DM populations. The accuracy of RQT_diff_ calculations hinges on precise identification of the QT interval. Although the QT intervals, regardless of T wave abnormalities on the ECG, were generally determined using MATLAB code with expert-defined rules (**Figure 3**), the QT intervals and RQT_diff_ values were found to be more reliably computed in subjects with normal T waves. In this study, 33.0% of participants in Group 2 (characterized by advanced age and T2DM) exhibited abnormal T wave morphologies on their ECGs (**Figure 1**), including T wave Types B (flattened), C (peaked), D (inverted), and E (other). In comparison, only 24.7% of the control group (Group 1) presented such abnormalities (**Table 2**). Previous studies have reported a prevalence of ECG abnormalities in diabetic populations ranging from 24.9% to 44.3%, depending on the cohort studied (29, 30). A recent investigation (31) has confirmed that ECG abnormalities are common in individuals with T2DM, with a prevalence of 29.1%. Moreover, a longitudinal study in Spain has demonstrated that the presence of ECG abnormalities predicted future cardiovascular events in patients with T2DM more accurately than traditional risk factors alone (32).

It is important to note that calculating and interpreting the QTc is critical, as the raw QT interval is influenced by the heart rate. Heart rate correction (QTc) was applied to mitigate this effect and ensure the comparability of QT interval measurements across different heart rates (26). However, unlike many previous studies (18, 26), which focused less on precise identification of the QT interval, this study emphasized the development of accurate methods to determine the QT interval for reliable RQT_diff_ calculations. Traditional HRV metrics have long been utilized to assess autonomic function; however, their sensitivity tends to diminish in populations with subtle autonomic dysfunction, such as the elderly and those with diabetes (33). This study demonstrated that, in healthy subjects (**Figure 4A**, Subject A), both RRI and RQT_diff_ sequences exhibited moderate fluctuations and followed a similar trend, with a strong correlation between the two (r = 0.996, *p* < 0.001; **Figure 4B**); however, in subjects with T2DM (**Figure 4A**, Subject C), the correlation between RRI and RQT_diff_ sequences was somewhat lower (r = 0.828, *p* < 0.001) but remained consistent. This reduction in correlation may stem from the relatively stable QTc across heartbeats, as RRI minus RQT_diff_ corresponds to the QTc. While RQT_diff_ reflects the electrical signal transmission within the heart, RRI encompasses both the contraction and relaxation phases. The diminished correlation and narrower range of variability in patients with T2DM suggest that diabetes may attenuate the relationship between these measures, potentially reflecting underlying autonomic dysfunction.

Furthermore, QTc values remained within a narrow range across all four subjects, aligning with findings from previous studies (34), which further supported the limited variability of RRI and RQT_diff_ in patients with diabetes. Notably, RQT_diff_ significantly enhanced the discriminatory power of entropy-based indices, such as MSE_SS_ and BEI (**Figures 4**, **5**). This enhancement was particularly useful in distinguishing patients with T2DM from healthy controls, indicating that RQT_diff_ captures aspects of autonomic regulation that are less apparent when using conventional RRI alone. These findings are consistent with previous studies on the complex nature of autonomic dysfunction in T2DM (35), where both the sympathetic and parasympathetic branches are impacted in ways that traditional HRV indices may not fully capture (36).

In the aging population, RQT_diff_ provided enhanced detection of autonomic dysfunction. As the ANS undergoes age-related changes, the QTc—which reflects ventricular repolarization—may offer deeper insights into the relationship between electrophysiological and autonomic mechanisms (37). Through incorporating RQT_diff_ into entropy analysis, a more nuanced assessment of HRV may be achieved, potentially facilitating earlier identification of individuals at risk for cardiovascular events. The clinical implications are substantial: increased sensitivity and specificity of RQT_diff_ in entropy-based HRV analysis could enhance diagnostic accuracy, improving the management and monitoring of elderly patients and those with T2DM. This approach could also be extended to other populations with autonomic dysfunction, broadening the clinical applicability of HRV analysis. Future research should explore the long-term effects of RQT_diff_-enhanced HRV analysis on patient outcomes, especially in relation to cardiovascular events and mortality. Additionally, standardized guidelines for the clinical application of RQT_diff_, including the development of normative values, are needed. The integration of RQT_diff_ into entropy-based HRV analysis represents a promising advancement, offering improved diagnostic capabilities for assessing autonomic dysfunction in aging and diabetic populations. In **Table 1**, while there was no statistically significant difference in RRI between Group 1 and Group 2, the longer QTc in Group 2 resulted in a statistically significant difference in RQT_diff_ between the groups (506.87 ± 73.87 ms vs. 471.79 ± 68.58 ms, *p* < 0.001). T2DM has been associated with prolonged QTcs in clinical settings (20), a phenomenon which was also observed in this study, as both aging and T2DM were linked to significant QTc prolongation (**Table 1**). Although this study initially proposed QTc as a potential substitute for RRI in ECG-based HRV assessment, significant differences were noted between Group 1 and Group 2 in MSE_SS_(QTc) and BEI(QTc) values (1.93 ± 0.05 vs. 2.01 ± 0.04 and 1.39 ± 0.02 vs. 1.48 ± 0.02, respectively; **Table 1**). Interestingly, this contrasts with the typically observed trend of reduced entropy values in Group 2. Upon closer examination, it was discovered that, while RRI did not differ significantly between the groups (422.71 ± 177.56 ms vs. 349.88 ± 143.81 ms, *p* = 0.003; **Table 1**), the difference between RRI and QTc (i.e., RQT_diff_) was statistically significant. This was evident from the trends in QTc and RQT_diff_ for all four subjects across varying ages and T2DM statuses, as shown in **Figure 4A**, leading to the realization that RQT_diff_ could emerge as a novel parameter for HRV assessment. This observation hints at its potential for clinical application (**Figures 4**, **5**), suggesting it may eventually influence clinical management, improve monitoring, and impact treatment outcomes in elderly and patients with diabetes.

The limitations of this study include the challenges in accurately identifying the Type E ECG T-wave, as shown in **Figure 1**. R-wave was preferred over the Q wave for QT interval measurement owing to its consistency as a reliable landmark, despite the Q wave technically marking the beginning of ventricular depolarization and the lack of investigation into medication use among Group 2 participants. Difficulties in accurately measuring the QT interval in abnormal ECG patterns may be addressed in future studies through the application of advanced artificial intelligence (AI)-based ECG analysis platforms, such as those developed using Python, which is widely adopted for machine learning and signal processing in biomedical research. Furthermore, as an exploratory clinical study, a formal prospective sample size calculation was not performed. However, the robust statistical significance observed in our cohort of 181 subjects, combined with the alignment of our sample size with established literature in ECG non-linear dynamics, suggests sufficient empirical power to detect the reported physiological differences. In addition, several potential confounding factors were not fully controlled in this study. Information regarding the duration of diabetes, detailed medication history, and coexisting comorbidities was not comprehensively available. These factors may influence autonomic function and HRV measures and could contribute to residual variability in the results. Future studies with more comprehensive clinical datasets are warranted to further validate the robustness of RQT_diff_-based analysis. What’s more, a potential limitation of this study is that manual correction of ECG features was performed by only two electrophysiologists, which may introduce observer-related bias. However, this was mitigated by independent blinded review and consensus-based adjudication. Finally, although this study employed Bazett’s formula for QTc correction, alternative correction methods, such as Fridericia’s and Hodges’ formulas (38), were also computed by the authors. Owing to space limitations, these results are not shown; however, ANOVA confirmed that there were no statistically significant differences among the correction methods.

## 5 Conclusions and future research

Accurate detection of the Q and T points in ECG signals remains a fundamental challenge, particularly because of the variability in T-wave morphology observed in clinical populations. This study successfully addressed this issue by developing and validating an automated algorithm capable of reliably identifying the Q and T points across diverse ECG patterns. Building on this foundation, we introduced a novel parameter—QTc–RR interval differential (RQT_diff_)—designed to enhance the sensitivity and specificity of entropy-based HRV assessments in ageing and T2DM populations. Our results demonstrated that RQT_diff_ provides a more comprehensive representation of autonomic regulation by integrating electrophysiological and mechanical components of cardiac function, may serve as a complementary tool for assessing autonomic dysfunction. Compared with traditional RRI-based metrics, RQT_diff_ exhibited superior discriminatory power, particularly in T2DM patients, where it revealed autonomic patterns not captured by conventional HRV indices. These findings suggest that RQT_diff_ may serve as a valuable adjunct for the early detection of autonomic dysfunction and could facilitate timely clinical interventions aimed at reducing cardiovascular risk in vulnerable populations.

Importantly, RQT_diff_ consistently improved the classification performance across multiple HRV indices, including MSE, BEI, and LHR. These improvements highlight the potential of RQT_diff_ as a complementary marker in entropy-based HRV analysis, offering enhanced diagnostic granularity when used alongside established parameters. Despite these promising results, further validation in larger and more diverse cohorts is warranted. Future research should explore the prognostic utility of RQT_diff_ in longitudinal studies, including its capacity to predict cardiovascular events, the progression of autonomic dysfunction, and overall mortality. Additionally, its application in populations with comorbid conditions—such as obesity, hypertension, and metabolic syndrome—should be investigated to evaluate its broader clinical relevance.

In the context of rapidly advancing healthcare technologies, the integration of AI holds great promise. Future studies should examine how AI-driven ECG analysis can improve the accuracy and robustness of RQT_diff_ extraction, particularly in ECGs with abnormal T-wave morphologies. Advanced signal processing and machine learning techniques may further refine RQT_diff_ as a diagnostic and prognostic tool in autonomic assessment.

## Data Availability

The raw data supporting the conclusions of this article will be made available by the authors, without undue reservation.
