# Supplementary material for: A novel QTc–RR differential biomarker for the early assessment of autonomic dysfunction in type 2 diabetes
Source: Front Endocrinol (Lausanne). 2026 Apr 30;17:1828837. doi: 10.3389/fendo.2026.1828837 (PMC13173515; doi:10.3389/fendo.2026.1828837)
Supplement: Supplementary file 1 [file DataSheet1.docx]

**Appendix A** Opcode-style Pseudocode Q and T-Point Detection Algorithm

BEGIN

1. LOAD ECG signal (Lead II), sampling rate = Fs

2. PREPROCESS:

a. Apply bandpass filter (e.g., 0.5–40 Hz) to remove baseline drift and noise

b. Normalize signal if needed

3. R-PEAK DETECTION:

a. Use Pan–Tompkins or derivative-based method

b. Store R_peak_indices = {r1, r2, ..., rn}

4. FOR each R_peak_index:

a. SET Q_SEARCH_WINDOW = 150 ms before R peak

b. Q_POINT = local minimum within Q_SEARCH_WINDOW using:

i. signal amplitude AND

ii. first derivative zero-crossing or max slope point

c. SET T_SEARCH_WINDOW = 200–500 ms after R peak (adaptive)

d. T_WAVE_SEGMENT = extract ECG segment in T_SEARCH_WINDOW

5. T POINT DETECTION (Morphology-Adaptive):

a. DETECT T wave peak = max(abs(T_WAVE_SEGMENT))

b. Estimate T_end using one of:

i. Threshold method: return-to-baseline point

ii. Tangent method: draw tangent at max slope of descending limb

iii. Derivative method: locate inflection or zero-slope point

c. IF multiple peaks or notches → use rule-based T-wave classification:

normal T wave, flattened T waves, peaked T waves, inverted T waves, and others.

6. STORE Q and T indices per beat

7. CALCULATE QT = T_index - Q_index (per beat)

8. APPLY QTc correction (e.g., Bazett or Fridericia)

9. OUTPUT QTc array or compute RQT_diff_ = QTc - RRI

END

**Appendix B** Opcode: T-Wave Morphology Classification BEGIN

INPUT: T_segment ← ECG samples within T-wave window (after R peak)

Fs ← Sampling frequency

1. NORMALIZE T_segment amplitude to zero baseline

2. DETECT PEAKS:

a. Find local maxima and minima within T_segment

b. Count number of peaks (n_peaks)

c. Record polarity (positive or negative)

3. CLASSIFY MORPHOLOGY:

IF n_peaks == 1 THEN

IF amplitude > threshold THEN

CLASSIFY as "Normal T wave"

ELSE

CLASSIFY as "Flattened T wave"

ELSE IF n_peaks == 2 THEN

IF first_peak_positive AND second_peak_negative THEN

CLASSIFY as "Peaked T wave"

ELSE IF first_peak_negative AND second_peak_positive THEN

CLASSIFY as "Inverted T wave"

ELSE

CLASSIFY as "Others：Irregular/Complex"

4. OPTIONAL: Use slope (1st derivative) or inflection points (2nd derivative)

to confirm peak transitions for ambiguous cases

OUTPUT: T_morphology_label ∈ {Normal T wave, Flattened T wave, Peaked T wave, Inverted T wave, Irregular}

END

**Appendix C: MATLAB Code for Q and T Point Detection from Lead II ECG**

% Q and T Point Detection Algorithm – Simplified MATLAB Version

% Input: ECG (Lead II signal), Fs = sampling frequency

% Output: Q_points, T_points, QT_intervals, QTc_intervals

function [Q_points, T_points, QTc_intervals] = detect_QT_points(ecg_signal, Fs)

% Step 1: Preprocessing

ecg_filtered = bandpass(ecg_signal, [0.5 40], Fs); % Bandpass filter

% Step 2: R-peak detection (Pan-Tompkins or similar)

[~, R_locs] = findpeaks(ecg_filtered, 'MinPeakHeight', mean(ecg_filtered)+0.5*std(ecg_filtered), ...'MinPeakDistance', round(0.6*Fs));

% Rough thresholding

% Initialize outputs

Q_points = zeros(size(R_locs));

T_points = zeros(size(R_locs));

QT_intervals = zeros(size(R_locs));

% Step 3: For each R peak, locate Q and T points

for i = 1:length(R_locs)

r = R_locs(i);

% Q-point search window (100–150 ms before R)

q_start = max(r - round(0.15 * Fs), 1);

q_end = r - round(0.02 * Fs); % Avoid including R peak

[~, q_rel_idx] = min(ecg_filtered(q_start:q_end));

Q_points(i) = q_start + q_rel_idx - 1;

% T-point search window (200–500 ms after R)

t_start = r + round(0.2 * Fs);

t_end = min(r + round(0.5 * Fs), length(ecg_filtered));

t_segment = ecg_filtered(t_start:t_end);

% Step 4: T-point detection (simple peak + tangent-based offset)

[~, t_peak_rel_idx] = max(abs(t_segment));

t_peak_idx = t_start + t_peak_rel_idx - 1;

% T-end estimation using return-to-baseline (zero-crossing)

baseline = mean(ecg_filtered(t_peak_idx:t_end));

t_end_idx = t_peak_idx;

for j = t_peak_idx:t_end

if abs(ecg_filtered(j) - baseline) < 0.02

t_end_idx = j;

break;

end

end

T_points(i) = t_end_idx;

% Step 5: QT and QTc interval

QT_intervals(i) = (T_points(i) - Q_points(i)) / Fs; % in seconds

end

% Step 6: Heart rate correction (Bazett’s formula)

RR_intervals = diff(R_locs) / Fs;

RR_intervals = [RR_intervals(1); RR_intervals]; % align length

QTc_intervals = QT_intervals ./ sqrt(RR_intervals);

end

**Appendix D: T-Wave Morphology Classification**

function T_morphology = classify_t_wave(T_segment, Fs)

% Classifies T-wave morphology

% INPUT:

% T_segment - ECG samples of the T-wave window (vector)

% Fs - Sampling frequency (Hz)

% OUTPUT:

% T_morphology - classification label (string)

% Normalize T-wave to baseline

T_segment = T_segment - mean(T_segment);

% Find peaks and troughs (positive and negative peaks)

[pks_pos, locs_pos] = findpeaks(T_segment);

[pks_neg, locs_neg] = findpeaks(-T_segment); % inverted signal for minima

pks_neg = -pks_neg; % restore actual values

% Combine peaks and sort by time

all_peaks = [pks_pos, pks_neg];

all_locs = [locs_pos, locs_neg];

[all_locs_sorted, idx] = sort(all_locs);

all_peaks_sorted = all_peaks(idx);

% Decision thresholds

AMP_THRESHOLD = 0.05 * max(abs(T_segment)); % 5% of max T-wave height

N_PEAKS = length(all_peaks_sorted);

% Classification logic

if N_PEAKS == 0 || max(abs(T_segment)) < AMP_THRESHOLD

T_morphology = 'Flat';

elseif N_PEAKS == 1

if all_peaks_sorted(1) > 0

T_morphology = 'Monophasic (+)';

else

T_morphology = 'Monophasic (-)';

end

elseif N_PEAKS == 2

if all_peaks_sorted(1) > 0 && all_peaks_sorted(2) < 0

T_morphology = 'Biphasic (+/-)';

elseif all_peaks_sorted(1) < 0 && all_peaks_sorted(2) > 0

T_morphology = 'Biphasic (-/+)';

elseif sign(all_peaks_sorted(1)) == sign(all_peaks_sorted(2)) ...

&& abs(all_locs_sorted(2) - all_locs_sorted(1)) < round(0.1 * Fs)

T_morphology = 'Notched';

else

T_morphology = 'Irregular';

end

elseif N_PEAKS > 2

T_morphology = 'Irregular';

else

T_morphology = 'Unclassified';

end

end
